# 

**Published:** 1879-07-20

**Authors:** 


### TABLE OF CONTENTS.

ORIGINAL AND SELECTED ARTICLES.

Spinal Wound—Tetanus—Death and Post Mortem, by A. B. Tadlock, A.M., M.D., 241. Brooklyn Anatomical Club—Report, by Geo. R. Fowler, M.D., 248. Vaccina Developed after Fourteen Years Incubation of the Virus, by Lindsay Johnson, M.D., 253. Buffalo Lithia Waters for Euræmia, Albuminuria of Pregnancy, Suppression of Urine in Yellow Fever, Menstrual Disorders, and Uric Acid Diathesis, by John W. Williamson, M.D., 255. Anesthetic for Children, 256.

ABSTRACTS AND GLEANINGS.

Effect of Posture in the Treatment of Strangulated and Incarcerated Hernia, 257. Euonymus Atropurpurens, 258. Opening the Mastoid Process by Surgical Procedure, 259. A Troublesome Parasite, 260. Propylamine Chlorid, 261. Puerperal Eclampsia; A Pleasant Remedy for Toothache, 262. Linseed and Oil in Skin Diseases, 263. Inverted Toe Nail; Salivation of Pregnancy, 264. Pyrogallic Acid in Internal Hemorrhage; Antidote to Poison; Poisoning by Glanders, 265. Arsenic as a Blood and Cardiac Tonic; Strangury from Blisters Prevented; Treatment of Hemorrhoids; Bryonia and Drosera for Hooping-cough, 266. Resection of the Infra-orbital Nerve; Cheap Disinfectant; Suberine in Excoriated Nipples, 267. Vaccination as a Preventive of other Diseases than Variola; Therapeutic Effects of Bryony; Phosphide of Zinc; Salicylic Acid Enemata in Dysentery, 268. Arrowroot for Infants; Iodide of Potassium in Vomiting; The Opium Habit; Treatment of Asthma, 269. Successful Cataract Extraction in an Unpromising Case; Cure of the Opium Habit; Bromide of Ammonium in too Frequent Menstruation; Cancer of the Womb Treated with Bromine Injections; Acute Glaucoma in a Woman 49 Years Old, 270.

SCIENTIFIC ITEMS.

A Voltaic Pencil; A Princely Trial of an Old Remedy; A Substitute for the Horse, 271, A Scientific Hoax; Physiological Action of Borax, 272.

PRACTICAL NOTES AND FORMULÆ.

Viburnum Prunifolium; Query; Pneumonia, 273. Hydrobromic Acid, 274. Simple Elixir; Syphilitic Phthisis; Dyspepsia, 275. Intermittent Fever; Hypodermic Injection of Ergotine; Quinia with Opium; Tinc. of Iodine in Cholera Infantum, 276.

EDITORIAL AND MISCELLANEOUS.

Editorial Notices; Book Notices, 277. Special Notices, 278. Southern Medical College Announcement, 279, 280.

